# Renal denervation in dialysis patients: long-term outcomes in a real-world setting

**DOI:** 10.1093/ckj/sfaf149

**Published:** 2025-05-13

**Authors:** Concetta Gangemi, Alessia Gambaro, Vittorio Ortalda, Domenico Tavella, Chiara Caletti, Simone Fezzi, Beatrice Bianco, Elisa De Tomi, Giovanni Gambaro, Flavio Luciano Ribichini, the GITIAR team, F Ribichini, D Tavella, V Ferrero, A Piccoli, A Gambaro, G Gambaro, C Gangemi, P Minuz, C Fava, R Scarsini, F Pizzolo, M Zamboni, F Fantin

**Affiliations:** Division of Nephrology, Department of Medicine, University of Verona, Verona, Italy; Division of Cardiology, Department of Medicine, University of Verona, Verona, Italy; Division of Nephrology, Department of Medicine, University of Verona, Verona, Italy; Division of Cardiology, Department of Medicine, University of Verona, Verona, Italy; Division of Nephrology, Department of Medicine, University of Verona, Verona, Italy; Division of Cardiology, Department of Medicine, University of Verona, Verona, Italy; Division of Nephrology, Department of Medicine, University of Verona, Verona, Italy; Division of Nephrology, Department of Medicine, University of Verona, Verona, Italy; Division of Nephrology, Department of Medicine, University of Verona, Verona, Italy; Division of Cardiology, Department of Medicine, University of Verona, Verona, Italy

**Keywords:** dialysis, renal denervation, resistant hypertension, transplantation, uraemia complications

## Abstract

**Background:**

Resistant hypertension is common in patients with end-stage kidney disease (ESKD) who are on dialysis. Experience with percutaneous renal denervation (RDN) in these patients is limited and generally has short follow-up periods, primarily focusing on blood pressure (BP) control. In particular, the effects of RDN on anaemia, chronic kidney disease–mineral and bone disorder (CKD-MBD) and dialysis tolerance remain unexamined.

**Methods:**

We report our experience with RDN in 14 dialysis patients [7 on haemodialysis (HD) and 7 on peritoneal dialysis (PD)] with a follow-up after RDN of 3–84 months. Six patients received kidney transplantation during follow-up.

**Results:**

BP and the number of antihypertensive drugs decreased (*P* = .008 and *P* = .018, respectively). RDN was equally effective in both HD and PD. In nearly all transplanted patients, BP normalized and the number of antihypertensive drugs was reduced after kidney transplantation. RDN did not alter treatment schedules for HD or PD and no episodes of acute intradialytic hypotension were reported. Additionally, there were no significant changes in treatments for anaemia or CKD-MBD. Only one patient had an RDN procedural complication.

**Conclusion:**

Our experience with RDN in dialysis patients suggests beneficial effects in both HD and PD. BP improved significantly, with no observed issues related to the typical clinical problems faced by these patients, such as anaemia, CKD-MBD and dialysis tolerance. Furthermore, in those patients who received a kidney transplant, we noted a further improvement in BP control.

## Graphical Abstract

**Graphical Abstract:**
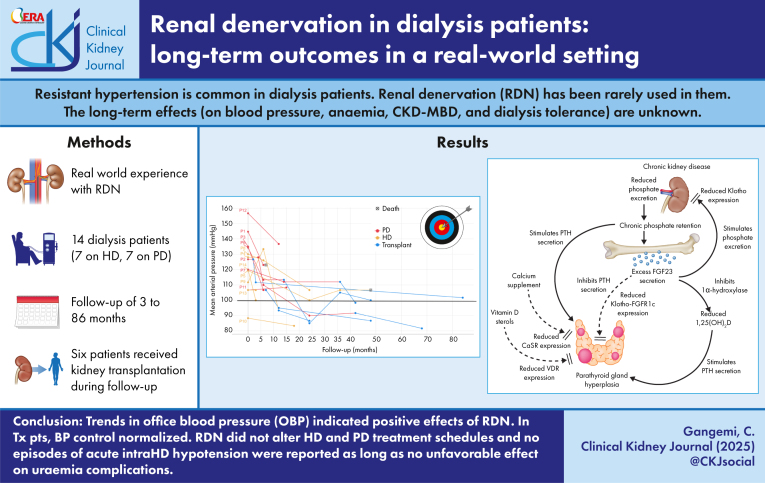

KEY LEARNING POINTS **What was known:**

- Resistant hypertension is prevalent in dialysis. The sympathetic activity is very high in ESKD. The autonomic system modulates erythropoietin and parathyroid hormone.
- Renal denervation (RDN) reduces blood pressure (BP) in resistant hypertension.
- The experience with RDN in dialysis is limited, primarily emphasizing short-term BP while the effects on anaemia and chronic kidney disease–mineral and bone disorder (CKD-MBD) are unknown.

**This study adds:**

- In dialysis patients, the hypotensive effect of RDN is achieved and remains persistent, without any adverse effects on dialysis tolerance, anaemia and CKD-MBD treatments.
- In the denervated dialysis patients, once transplanted, BP control further improves.
- The RDN procedure is safe in dialysis patients both peri-procedurally and in the long-term.

**Potential impact:**

- RDN seems to be a valuable and safe option for the treatment of severe hypertension in dialysis patients.
- It allows downsizing the pharmacological treatment and allows safe transplantation.

## INTRODUCTION

Hypertension that is resistant to treatment is frequently observed in patients with end-stage kidney disease (ESKD) undergoing dialysis. Studies have shown that the prevalence of resistant hypertension in this population ranges from 18% to 42% [1]. These patients experience elevated mortality rates primarily due to cardiovascular (CV) diseases [2], and for this reason, they may be excluded from transplantation lists until their blood pressure (BP) is controlled. In chronic kidney disease (CKD) patients, sympathetic activity progressively increases with the severity of renal dysfunction, reaching its highest levels in ESKD [3]. This overactivity is a major driver of hypertension in both CKD and ESKD. Additionally, the autonomic system, beyond its role in regulating BP and cardiac rhythm, also modulates the secretion of erythropoietin and parathyroid hormone [4–6].

Renal sympathectomy, obtained with percutaneous renal denervation (RDN), has been developed to reduce BP in resistant and uncontrolled hypertension. After the first randomized trial on percutaneous catheter–based RDN in 2010 [7], several others have been performed, and in November 2023, the US Food and Drug Administration approved this technique as an adjunctive treatment option when lifestyle changes and medication do not result in adequate BP control [8]. Until now, CKD with a glomerular filtration rate (GFR) <40 ml/min/1.73 m^2^ has been an exclusion criterion of RDN trials. A European consensus statement concluded that it is not advised to perform RDN in kidney transplant recipients or patients with severely impaired kidney function (Kidney Disease: Improving Global Outcomes stage G4 and G5) or who require haemodialysis (HD) [9]. The recent European Society of Cardiology guidelines state that RDN is not recommended in patients with an eGFR <40 ml/min/1.73 m^2^ [10]. Thus the experience of CKD patients treated with RDN is limited to a few small studies of hypertensive CKD patients [11–13]. Even more limited is the experience in ESKD treated with HD, accounting for only 39 published patients—four case reports [14–17] and five small series or cohorts [18–22] (Supplementary Table S1). These reports had a short follow-up of 6–24 months and the main emphasis was on BP control. Clinical issues related to HD tolerance and treatment *per se* and CV events were only scantly [21] or not at all analysed [18–20, 22]. Furthermore, the short follow-up prevented the possibility of investigating the impact of RDN on the whole clinical ‘journey’ of a renal patient, which includes changes in dialysis techniques and transplantation. Moreover, the experience of RDN in peritoneal dialysis (PD) patients is even smaller than within HD, accounting for only five patients [19]. Since 2015, 14 of our ESKD patients on dialysis have undergone RDN. In this article we analyse the clinical course in this case series, emphasizing not only the effect on BP, but also on the main clinical issues faced by dialysis patients, namely anaemia, chronic kidney disease–mineral and bone disorder (CKD-MBD), dialysis tolerance and residual diuresis.

## MATERIALS AND METHODS

RDN has been performed in 14 of our ESKD patients on dialysis therapy and clinically stable for at least 6 months (7 HD patients and 7 PD patients). All HD patients received three 4-h dialysis sessions per week. In October 2024, the follow-up ranged from 3 to 84 months and eight of them had a follow-up >2 years; six patients received a kidney transplant. Patients on HD or PD with resistant hypertension were proposed for RDN only after verifying with bioimpedance that they were not overhydrated and that the depuration indexes were in accordance with guidelines. Each patient was evaluated jointly for RDN by the dedicated multidisciplinary team of the hospital (the GITIAR team) composed of nephrologists, cardiologists and hypertension specialists. The selection of patients with resistant hypertension for RDN was done following a flow chart like the one suggested in the position paper of the Italian Society of Arterial Hypertension [23] (Supplementary Fig. S1).

The protocol includes clinical evaluation and imaging of the renal arteries. RDN is not executed in patients who have, at renal angiogram performed during the RDN procedure, a haemodynamically significant renal artery stenosis (stenosis diameter >70%) with the demonstration of a significant (>20 mmHg) trans-lesional pressure gradient [24] or a renal artery diameter <2 mm.

The protocol was approved by the local ethics committee (CESC-2361) and by the Department of Health of the Veneto Region [13]. All patients signed an informed consent and were enrolled in the Global Symplicity Registry (GSR) [25]. The bilateral RDN procedure has been previously described [13]. A unipolar catheter (Symplicity Flex; Medtronic, Minneapolis, MN, USA) was used in one patient (patient 1), while the others were treated with a tetrapolar catheter (Symplicity Spyral; Medtronic). According to the protocol, follow-up visits by the cardiologist are scheduled at 3, 6 and 12 months post-procedure. Office BP (OBP) was measured at each visit (Supplementary Table S2). Assessment of medication intake was determined through direct questioning. The protocol also includes the assessment of 24-h ambulatory BP monitoring; however, not all patients agreed to undergo it at some of the scheduled times. Furthermore, during the follow-up after the first year, only OBP measurements were taken. Therefore, for the sake of consistency, only OBP values are presented. OBP is the mean of three recordings taken while sitting after a 5-minute rest using a validated sphygmomanometer. In HD patients, OBP was measured before the mid-week dialysis session. Other clinical data in the first 12 months following RDN were collected as part of routine clinical practice at each dialysis session or outpatient visit during follow-up, conducted by the nephrologists responsible for the patients. The medical information regarding the patients’ clinical status, OBP, medication regimen, adherence to antihypertensive treatment, episodes of intradialytic hypotension, changes in the dialysis schedule or in dialysis technique, residual diuresis (qualitatively reported but not quantified through 24-h urine collection), anaemia management and treatment for CKD-MBD, cardiac arrhythmias and CV events was obtained from clinical records and through a questionnaire distributed to the nephrologists overseeing the patients (Supplementary Material). In the questionnaires, nephrologists were asked to provide a clinical assessment of these aspects. Statistical analysis of the trajectory of mean arterial pressure and the number of medications during follow-up was performed using a linear mixed effects model with a random intercept for each patient to account for repeated measurements. Fixed effects included time, clinical status (HD, PD, transplant), age at baseline, sex and the presence of diabetes. Additionally, a further analysis was conducted excluding patients with only two observations, to verify the robustness of the results. The open-source lme4 package in R version 4.1.1 (R Foundation for Statistical Computing, Vienna, Austria) was used.

## RESULTS

### Patient characteristics

The patients’ ages ranged from 21 to 81 years. Apart from two patients (ages 78 and 81 years), the others were <56 years of age. Sex, presence of diabetes, causes of ESKD, changes in dialysis technique and overall follow-up are shown in Table 1. In one patient (patient 0), who had good BP control with two drugs, RDN was not performed for resistant hypertension but for a very high CV risk.

**Table 1:** Demographic and general clinical data of dialysis patients who underwent RDN.

Patient	Sex	Age (years)^a^	ESKD cause	Diabetes	Dialysis type at RDN	Changed RRT during follow-up	Overall follow-up (months)
1	M	54	Diabetic nephropathy	Yes (type 2)	HD		7^b^
2	M	41	IgAN	No	HD	Tx	84
3	M	21	IgAN	No	HD	Tx	48
4	M	44	Single kidney. Unknown	No	PD	HD, Tx	68
5	M	81	Hypertensive nephrosclerosis	No	PD		48^b^
6	M	32	Hypertensive nephrosclerosis	No	HD	Tx	42
7	M	27	Unknown	No	HD	Tx	42
8	F	50	Hypertensive nephrosclerosis	No	PD		24
9	F	46	Hypertensive nephrosclerosis	No	PD	Tx	24
10	M	78	Hypertensive nephrosclerosis	No	PD		18
11	M	56	IgAN	Yes	HD		15
12	M	53	Diabetic nephropathy	Yes (type 1)	HD		12
13	M	49	Hypertensive nephrosclerosis and NSAID abuse	No	PD		3
14	M	42	Fibrillary glomerulopathy	No	PD		3

a At RDN.

^b^Deceased.

IgAN: immunoglobulin A nephropathy; NSAID: non-steroidal inflammatory drugs; RRT: renal replacement therapy; Tx: kidney transplantation.

### Effect on BP and treatment

**Figure 1:**
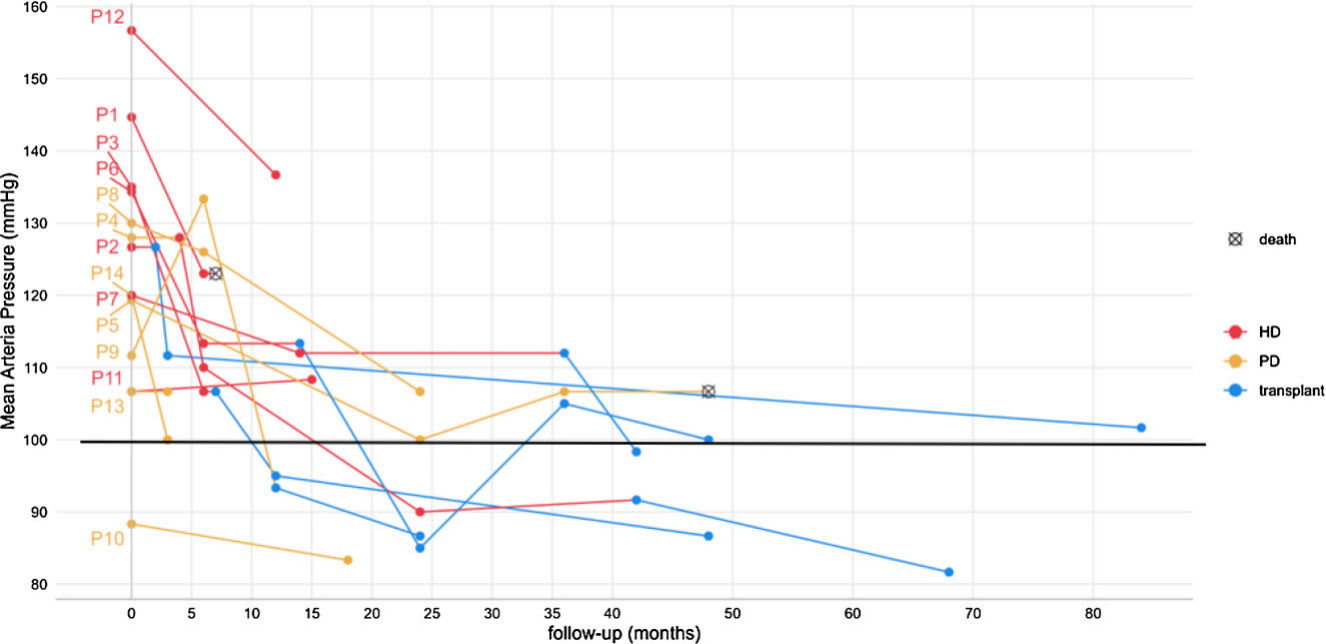
Time course of mean OBP after RDN in dialysis patients. Mean OBP is [(systolic BP + 2 diastolic BP)/3]. The overall follow-ups of the 14 patients are shown; the red lines refer to HD, the yellow lines refer to PD and the blue lines refer to kidney transplantation. The horizontal black line corresponds to an OBP of 140/80 mmHg.

Fig. 1 shows the time course of mean OBP [(systolic BP + 2 diastolic BP)/3] in each patient during the entire follow-up period. Supplementary Figs. S2–S4 show the number of antihypertensive agents at different time points for each patient.

**Figure 2:**
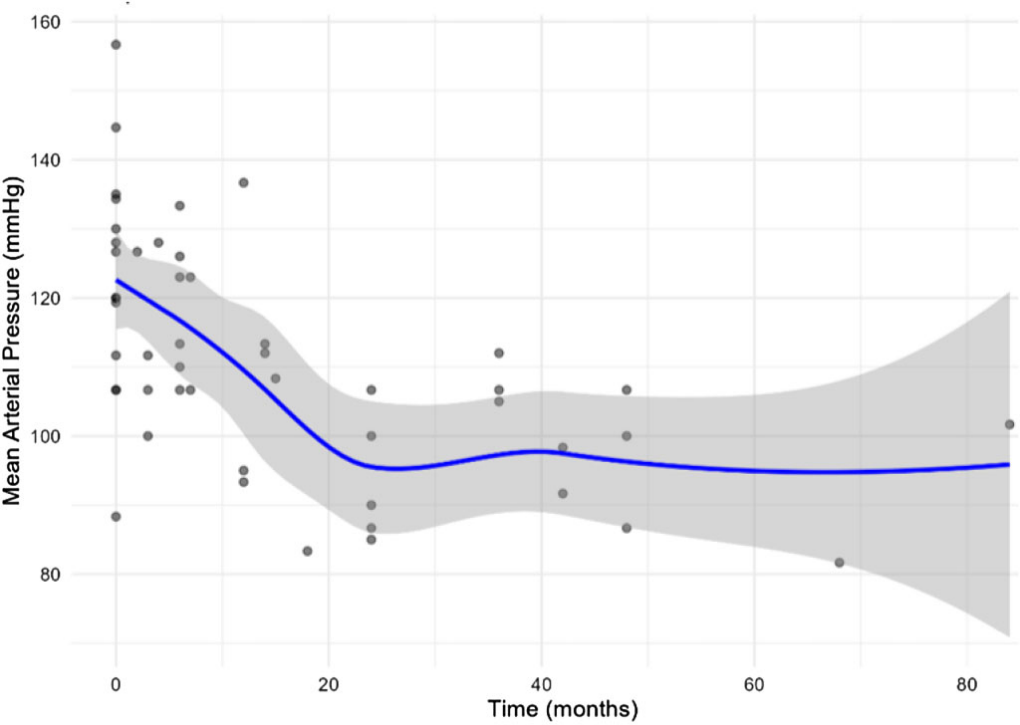
Trajectory of mean OBP after RDN during the entire follow-up period. Mean OBP decreased by 0.3 mmHg/month (*P* = .008). The shadow area shows confidence intervals.

**Figure 3:**
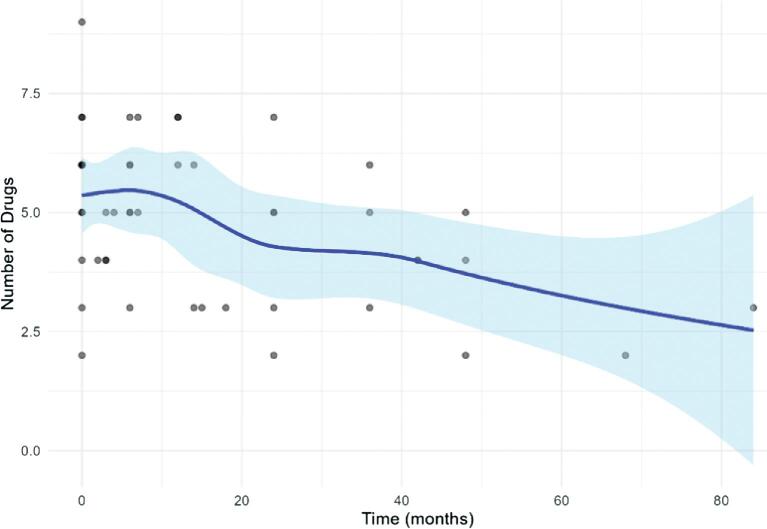
Trajectory of the number of drugs after RDN during the entire follow-up period. The number of drugs decreased by 0.025 pills/month (*P* = .018). The shadow area shows confidence intervals.

Considering the entire follow-up period, the analysis showed that after RDN there was a significant reduction over time in mean OBP (−0.3 mmHg/month; *P* = .008) and in the number of antihypertensive medications (−0.025 medications/month; *P* = .018), after adjusting for all covariates [clinical status (HD, PD or transplant), age at baseline, sex and diabetes] (Figs. 2 and 3). However, it is clear that the OBP reduction occurred in the initial 24 months of follow-up (≈30 mmHg; adjusted for the above covariates −1.27 mmHg/month; *P* = 2.43e-06), while it remained substantially stable in the rest of the follow-up period. A reduction in the pill burden was observed across almost all patients.

Focusing solely on the dialysis follow-up, patients on HD showed a trend towards a greater reduction in mean OBP compared with PD patients (−1.46 mmHg/month versus 0.52 mmHg/month, respectively; *P* = .08443, not significant). Notably, in transplanted patients there was a further reduction in OBP (0.22 mmHg/month; *P* = .00374), with nearly all patients almost normalizing their BP (Supplementary Fig. S3).

### Effect on the clinical issues of dialysis patients

The treatment schedules for HD and PD were not affected by RDN, and episodes of acute intradialytic hypotension were not observed during the follow-up of HD patients (Table 2). Only one patient had to be switched from PD to HD, and this was due to severe peritonitis, which was independent of BP control and RDN. Residual diuresis in dialysis patients was not significantly altered by RDN (Table 2). The nephrologists responsible for the clinical follow-up of patients reported no issues or variations in anaemia and CKD-MBD parameters and treatment, aside from a few cases (Tables 2 and 3). Therefore, there was no indication of any worsening or improvement trend in these clinical issues after RDN.

**Table 2:** Clinical events during follow-up of dialysis patients who underwent RDN.

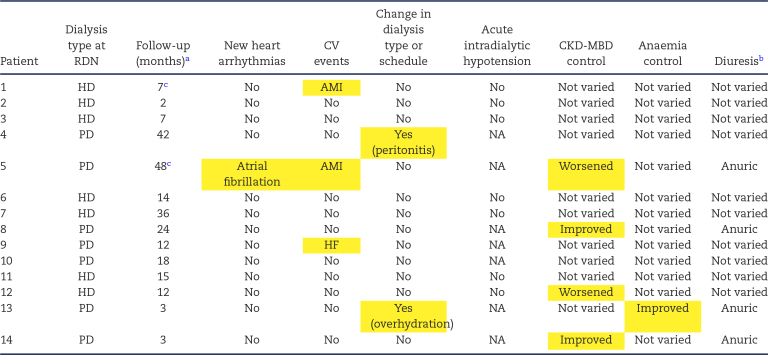

a Follow-up on dialysis after RDN.

b On dialysis.

^c^Deceased.

Events and changes that occurred during the follow-up are highlighted in yellow.

AMI: acute myocardial infarction; HF: heart failure. All anuric patients were anuric at the time of RDN.

**Table 3: Clinical events during the follow-up after transplantation in d:** ialysis patients who underwent RDN and subsequently received a transplant.

Patient	Follow-up (months)^a^	BP control^b^	New heart arrhythmias	CV events	CKD-MBD control	Anaemia control
2	82	Improved	No	No	Not varied	Not varied
3	41	Improved	No	No	Not varied	Not varied
4	26	Improved	No	No	Not varied	Not varied
6	34	Improved	No	No	Not varied	Not varied
7	6	Improved	No	No	Not varied	Not varied
9	12	Improved	No	No	Not varied	Not varied

a After transplantation.

b Compared with BP control on dialysis.

### Safety of RDN

One patient, an 81-year-old man, and the oldest person in the study, developed chronic atrial fibrillation. He subsequently died from acute myocardial infarction 48 months after RDN. Another patient died after only 7 months post-RDN, but from an unrelated cause. Specifically, he developed pneumonitis and sepsis following a femoral neck fracture and during hospitalization he experienced a non-ST-elevation myocardial infarction.

Regarding the periprocedural safety of the RDN treatment, no complications were observed except in one patient who developed a femoral pseudo-aneurysm.

## DISCUSSION

One of the primary contributors to hypertension in patients undergoing dialysis is sodium retention and an increase in blood volume [1, 26]. Other contributors to hypertension include endothelial dysfunction, activation of the renin–angiotensin–aldosterone system and heightened activity of the sympathetic nervous system. The influence of sympathetic nerve factors is underscored by findings that shed light on how noradrenergic activation may occur [27]. For instance, the native kidneys can transmit afferent nerve signals to the central nervous system, which can trigger sympathetic overactivity. Moreover, sympathetic nervous system activity tends to increase as CKD progresses and afferent renal nerves may exert an excitatory effect on central sympathetic output in response to renal injury [3]. Ultimately, both renal sympathetic efferent and afferent nerves significantly impact the onset, persistence and worsening of high BP commonly seen in ESKD patients, frequently resulting in resistant hypertension [26]. This is the rationale that has led to the use of RDN for the treatment of resistant hypertension, a treatment that might be highly effective in patients with ESKD. However, few studies conducted on small series or cohorts of dialysis patients have addressed the effect of percutaneous RDN on resistant hypertension. They have shown that BP and/or the number of antihypertensive medications generally improved. However, all these studies had a relatively short duration, lasting no longer than 6–24 months (Supplementary Table S1). Patients with ESKD often have a complex and longer clinical history, sometimes transitioning between the two forms of dialysis (HD or PD) or to and from transplantation. While the mid-term effects of RDN on BP control have been investigated, any effects on anaemia and CKD-MBD treatments and dialysis tolerance have not been examined.

We treated 14 dialysis patients with RDN from 2015 to October 2024, of which 7 were on PD. On average, we treat 220 HD patients and 45 PD patients in our wards. Thus the ecological prevalence of RDN is 3.6% for HD and 15.5% for PD. This finding may seem surprising; however, the burden of hypertension in PD patients is like that observed in HD patients. According to the Italian Cooperative Peritoneal Dialysis Study Group, in a cohort of 504 PD patients, the prevalence of hypertension was 88%, with 79% of drug-treated hypertensive patients having uncontrolled clinic BP [28]. Furthermore, after the initial 6–12 months of improved BP following the initiation of PD, a progressive deterioration in BP control occurs [29].

As mentioned, sodium retention and increased fluid volume are the main contributors to hypertension in patients undergoing dialysis [25]. This is particularly relevant for PD patients, as volume overload is common due to liberal fluid intake and the loss of residual renal function. Studies have demonstrated that alleviating volume overload by eliminating excess sodium and adjusting target dry weight can lead to improvements in BP for those receiving dialysis [30, 31]. However, during the selection phase of dialysis patients for eligibility for RDN, we specifically focused on excluding overhydration as a factor contributing to resistant hypertension, particularly in PD patients. The sustained effect of RDN on BP in PD patients over time supports the view that this effect is not dependent on any modification of volume load. 

Similar to other studies, we observed a favourable effect of RDN on BP and on the number of medications (Figs. 2 and 3). Patient 12 did not show any improvement, likely due to poor adherence (Fig. 1).

Experimental and clinical studies have demonstrated the role of adrenergic activation in the risk of ventricular hypertrophy and arrhythmogenesis [32–34]. Clinical studies have demonstrated that RDN reduces the incidence of atrial fibrillation and CV events, even in the absence of a significant antihypertensive effect [35]. With this background and the aim of reducing the elevated CV risk, we treated patient 10 with RDN, the only patient who did not present with resistant hypertension.

Nevertheless, autonomic regulation of the heart and circulation undergoes significant and complex changes in individuals with CKD and ESKD, affecting both parasympathetic and sympathetic control over the heart and peripheral blood vessels. We were concerned that, because of such derangements, acute intradialytic hypotensive episodes, major cardiac arrhythmias and fatal and non-fatal CV events might occur in dialysis patients. However, during follow-up after RDN, patients did not experience any of these conditions. In HD patients, episodes of acute intradialytic hypotension were not observed during the follow-up period (Table 2). We recorded only two deaths after 7 and 48 months (patient 1 due to sepsis and patient 5 due to acute myocardial infarction), a number lower than the expected 40% 5-year mortality rate observed in dialysis patients [36]. Although BP reduction after RDN was associated with a lower incidence of long-term CV events in patients with resistant hypertension compared with non-responders [37], we believe that the low number of deaths in our cohort is primarily due to selection bias, as only patients with better survival prospects and/or younger age were selected for the RDN procedure.

Renal nerves influence fluid volume regulation through renal sympathetic efferent nerves by increasing the activity of the systemic renin–angiotensin system, renin secretion and sodium absorption [38]. Thus RDN may impair fluid control in some way. We explored this possibility by evaluating whether diuresis, which serves as a proxy for fluid control and residual renal function, was altered after RDN. However, no variations were observed.

The sympathetic nervous system is involved in bone metabolism, as evidenced by the high bone mass phenotype observed in mice with low sympathetic activity [39]. Notably, in dialysis patients, a connection has been identified between sympathetic hyperactivity due to uraemia and CKD-MBD [5, 6]. Therefore, we investigated whether RDN had any effect on secondary hyperparathyroidism and the treatment of CKD-MBD. Our clinical experience did not reveal any negative trends associated with RDN (Tables 2 and 3).

Since erythropoiesis is impaired when the kidney is denervated due to reduced erythropoietin production in response to hypoxia [4], we also examined whether there were any clinical issues with treatment for anaemia. Again, RDN showed no impact on this aspect (Table 2).

During follow-up, six dialysis patients received kidney transplants. The significant BP improvement after RDN allowed them to re-enter the kidney transplant list, from which they had previously been excluded for the high CV and peri-procedural bleeding risks related to poorly controlled hypertension. 

In these patients, CKD-MBD, anaemia and its treatment did not require any special attention (Table 3). Interestingly, they experienced further improvements in OBP and required fewer antihypertensive medications over time (Supplementary Figs. S3 and S6) despite receiving immunosuppressive agents such as corticosteroids and tacrolimus, which are typically associated with increased BP [38].

Previous research has shown that restoring renal function through kidney transplantation does not lead to a decrease in muscle sympathetic nerve activity (MSNA) if the native kidneys remain in place. In fact, only a kidney transplant—where RDN is inherently part of the surgical procedure—combined with bilateral nephrectomy can achieve the desired normalization of sympathetic nerve activity [41, 42].

To our knowledge, there has been only one report of an HD patient with RDN receiving a kidney transplant [18]. Like our observations, this patient also experienced an improvement in BP and a reduced need for antihypertensive medications following transplantation. Interestingly, this patient exhibited only a slight decrease in MSNA after RDN of the native kidneys, whereas this activity progressively normalized post-transplantation. It has been suggested that the functional nephrectomy achieved through bilateral RDN—particularly the reduction of afferent sympathetic signalling from the diseased native kidneys—played a significant role in this series of events [18].

Important limitations of this study are that it is a single-centre retrospective analysis without a control group for comparison. These factors inherently restrict the generalizability and causal interpretation of the results.

In conclusion, our experience with RDN performed on dialysis patients suggests beneficial effects in both HD and PD. BP control was significantly improved and we did not observe any issues related to the typical clinical problems faced by these patients, such as anaemia, CKD-MBD and HD intolerance. Furthermore, in those patients who received a kidney transplant, we observed an additional improvement in BP control. However, the small number of patients in this uncontrolled, retrospective study prevents us from drawing firm conclusions, which could be provided by a trial and/or analysis of data from a registry.

## Supplementary Material

sfaf149_Supplemental_File

## Data Availability

Data are available upon request.
