# Supplementary material for: Renal denervation in dialysis patients: long-term outcomes in a real-world setting
Source: Clin Kidney J. 2025 May 13;18(6):sfaf149. doi: 10.1093/ckj/sfaf149 (PMC12209812; doi:10.1093/ckj/sfaf149)
Supplement: sfaf149_Supplemental_File [file sfaf149_Supplemental_File.docx]

**Supplementary material**

**Table**

**Table S1**

**RDN in dialysis patients: data from the literature**

|  | **#HD pts** | **#PD pts** | **#Tx pts** | **Follow-up (months)** |
| --- | --- | --- | --- | --- |
| Di Daniele N et al. Nephrol Dial Transplant 2012 (14) | 1 |  |  | 1 |
| Prochnau D et al. Int J Cardiol 2012 (15) | 1 |  |  | 3 |
| Ott C et al. J Clin Hypertens 2012 (16) | 1 |  |  | 6 |
| Schlaich MP et al. Int J Cardiol 2013 (18) | 9 |  | 1* | 12 |
| Hoye NA et al. Kidney Int Rep 2017 (19) | 6 | 3 |  | 12 |
| Pietilä-Effati PM et al. Am J Case Rep 2018 (20) | 2 | 2 |  | 12-24 |
| Ott C et al. Clin Exp Nephrol 2019(21) | 6 |  |  | 6 |
| Scalise F et al. J Hypertens. 2020 (22) | 12 |  |  | 12 |
| Mazza A et al. Curr Hypertens Rep 2023 (17) | 1 |  |  | 6 |

Legend: #, number; *, this patient, one of nine, received a kidney transplant 4 months after the RDN.

**Table S2**

**OBP in the 6 months before RDN**

| **# Patient** |  |
| --- | --- |
| **1** | 150/100, 160/108, 170/110, 200/110, 224/105 mmHg |
| **2** | 160/100, 165/105, 173/109, 160/110 mmHg |
| **3** | 160/105, 170/110, 174/102, 205/100 mmHg |
| **4** | 169/121, 180/110, 170/100, 156/114 mmHg |
| **5** | 190/90, 174/92 mmHg |
| **6** | 150/105, 160/100, 160/110,175/110, 161/121 mmHg |
| **7** | 160/100, 175/102, 178/95, 175/95, 150/105 mmHg |
| **8** | 160/90, 160/100, 170/110 mmHg |
| **9** | 184/116, 175/95, 170/100 mmHg |
| **10** | 150/90, 145/90 mmHg |
| **11** | 180/100, 185/95,173/90, 170/98 mmHg |
| **12** | 155/95 e 165/101,185/105, 172/112, 220/125 mmHg |
| **13** | 170/90, 170/100 mmHg |
| **14** | 160/100, 160/95, 160/100 mmHg |

OBP was determined in a non-dialysis day.

**Figures**

Fig. S1

Title: **Patient’s selection flow chart**


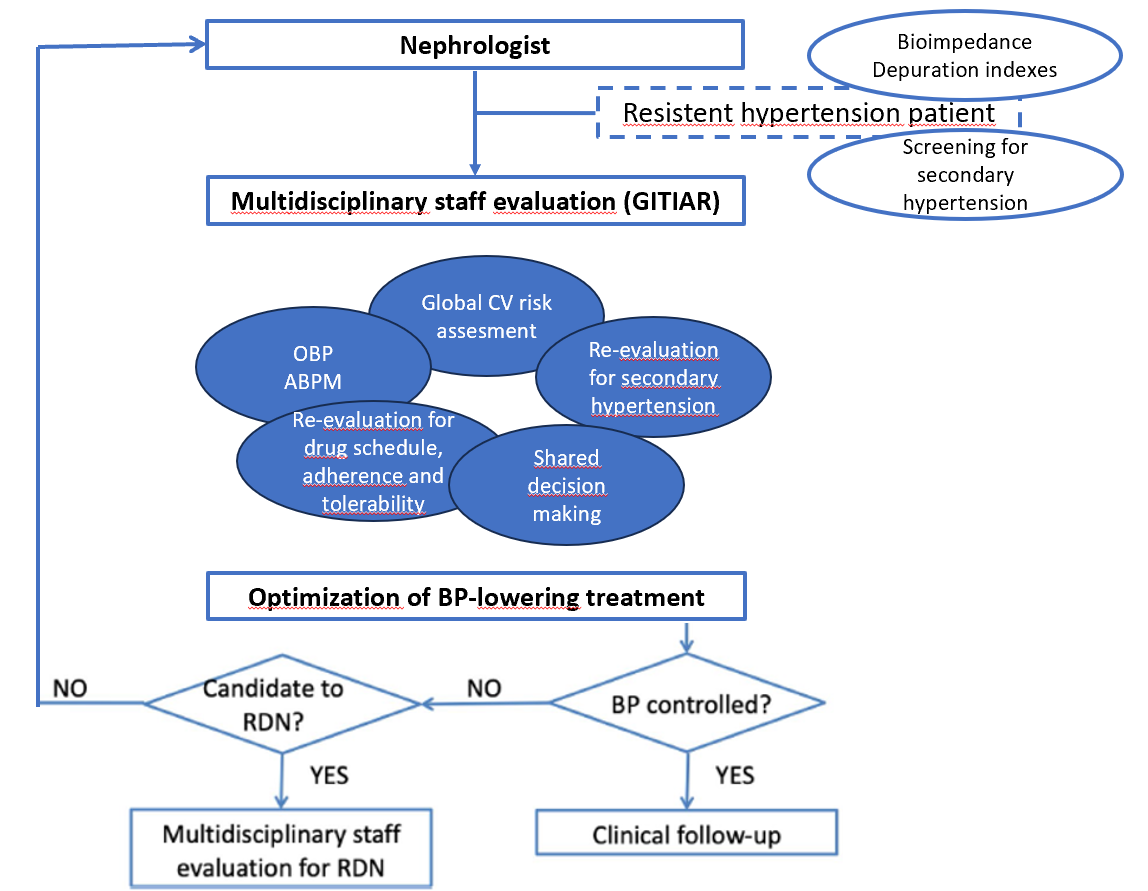


Fig. S2

Title: **Time course of mean OBP after RDN in HD patients.**

**
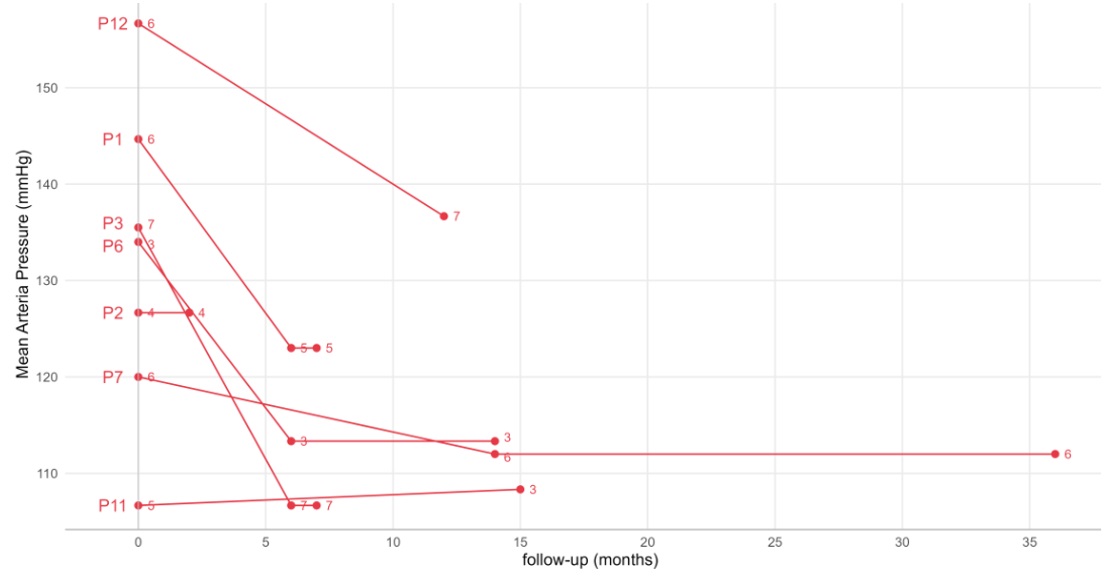
**Legend: The follow-ups during the dialysis period for the 7 HD patients are shown; the red lines indicate HD, while the blue lines represent kidney transplantation. The number of antihypertensive agents at different time points is reported.

Fig. S3

Title: **Time course of mean OBP after RDN in PD patients.**


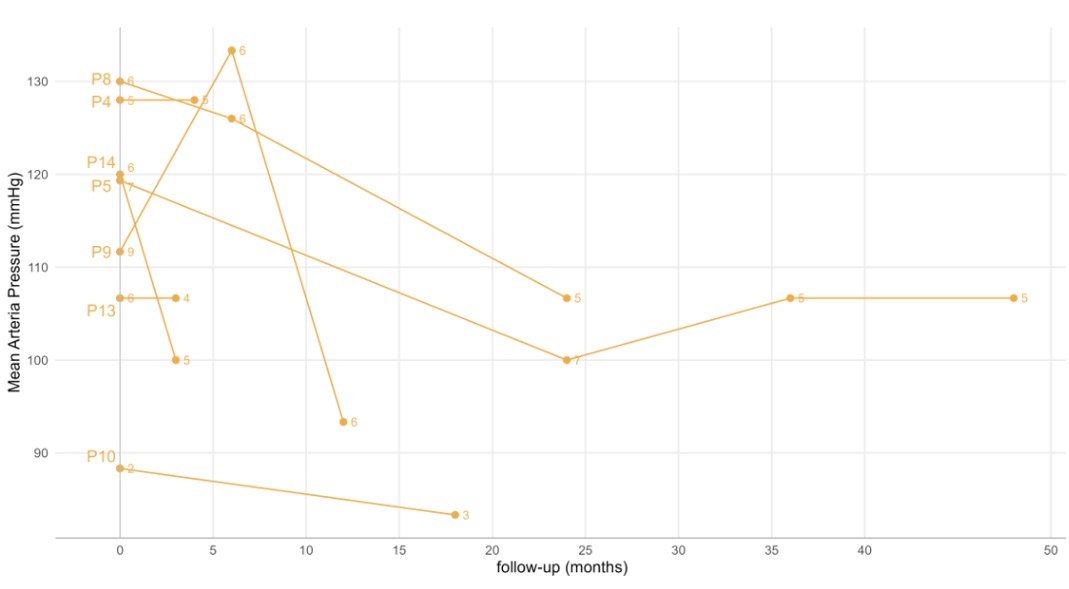

Legend: The follow-ups during the dialysis period for the 7 PD patients are shown. The number of antihypertensive agents at different time points is reported.

Fig. S4

Title: **Time course post-RDN of mean OBP after kidney transplantation.**

**
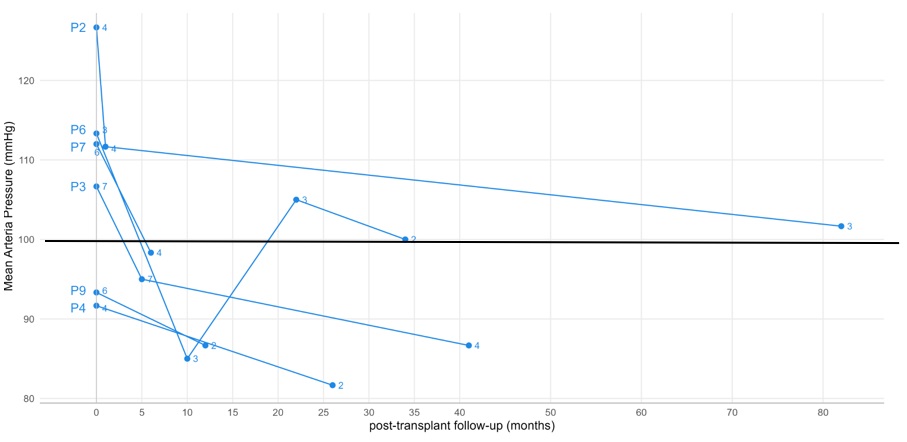
**

Legend: The follow-ups during the transplant period for the six transplanted patients are shown. The number of antihypertensive agents at different time points is reported. The horizontal black line corresponds to an OBP of 140/80 mmHg.

**EFFECT OF RENAL DENERVATION IN DIALYZED PATIENTS**
This questionnaire refers to the detection of new symptoms/manifestations or changes in intensity and treatment that occurred after denervation (RDN) compared to the period before denervation.

**Patient name: __________________________________**

Cause of ESKD: ________________________ Diabetes YES / NO Dialysis Vintage^[[1]](#footnote-1)^ ____years

Subsequent to the denervation (date of RDN ……………….)

BLOOD PRESSURE CONTROL IS

- Not varied
- Improved
- Worsened

What therapy is she/he taking at the last available check-up (please specify)?_________________

What's the pressure in the most recent available check? ________mmHg

Has there been a development of new heart rhythm alterations?

- NO
- YES
  - Tachyarrhythmias (e.g., new diagnosis of atrial fibrillation, atrial tachycardia, ventricular tachycardia)
  - Bradyarrhythmias (did they need a pacemaker or defibrillator?

Have cardiovascular events occurred?

- NO
- YES
  - Which (please specify)? _______________________________________________________

If he/she was in HD, did he/she change the type of dialysis?

- NO
- YES
  - Which ? ____________________________________________________________________
  - Reason? ___________________________________________________________________

If he/SHE was in HD, did he/she develop intolerance to haemodialysis.?

- NO
- YES
  - Which manifestations of intolerance (intradialytic hypotension, others, please specify)"? _________________________________

If HE/SHE WAS IN PD

- He/She has shifted to haemodialysis (please specify the reason)_____________________________
- The dialysis schedule is unchanged.
- The dialysis schedule is changed. (PLEASE specify) _____________________________________
  - Reason? ___________________________________________________________

Residual diuresis is (for both patients, on HD or PD)

- Not varied
- Worsened

CKD-MBD control^[[2]](#footnote-2)^ is

- Not varied
- Improved (please specify) ? _______________________________________________________
- Worsened (please specify) ? _______________________________________________________

ANAEMIA control^[[3]](#footnote-3)^ is

- Not varied
- Improved (please specify) ? _______________________________________________________
- Worsened (please specify) ? _______________________________________________________

COMMENTS _______________________________________________________________________

_______________________________________________________________________­­­­­­­­­­­­­­_____________

**EFFECT OF RENAL DENERVATION IN DIALYZED PATIENTS WHO HAVE BEEN SUBSEQUENTLY TRANSPLANTED**

**Patient name: __________________________________**

Do you observe specific problems regarding:

BLOOD PRESSURE CONTROL

- NO
- YES
  - Which (please specify) ? _______________________________________________________

What therapy is she/he taking at the last available check-up (please specify)?_________________

What's the pressure in the most recent available check? ________mmHg

CKD-MBD control^[[4]](#footnote-4)^

- NO
- YES
  - Which (please specify) ? _______________________________________________________

anaemia control^[[5]](#footnote-5)^

- NO
- YES
  - Which (please specify)? _______________________________________________________

Has there been a development of new heart rhythm alterations after transplantation?

- NO
- YES
  - Tachyarrhythmias (e.g., new diagnosis of atrial fibrillation, atrial tachycardia, ventricular tachycardia)
  - Bradyarrhythmias (did they need a pacemaker or defibrillator?

Have cardiovascular events occurred after transplantation?

- NO
- YES
  - Which (please specify)? _______________________________________________________

COMMENTS­­­­­­­­­­­­­_________________________________________________________________________

________________________________________________________________________

1. on the date of denervation [↑](#footnote-ref-1)
2. It refers to a comprehensive assessment of treatment and outcomes in light of the biochemical parameters (calcium, phosphorus, PTH, etc.) [↑](#footnote-ref-2)
3. It refers to a global assessment of the treatment (erythropoietin, iron, etc.) and the results in light of the biochemical parameters (haemoglobin levels, etc.). [↑](#footnote-ref-3)
4. It refers to a comprehensive assessment of treatment and outcomes in light of the biochemical parameters (calcium, phosphorus, PTH, etc.) [↑](#footnote-ref-4)
5. It refers to a global assessment of the treatment (erythropoietin, iron, etc.) and the results in light of the biochemical parameters (haemoglobin levels, etc.). [↑](#footnote-ref-5)
